# Exploiting Synthetic Lethality of PRMT5 for Precision Treatment of MTAP-Deficient Glioblastoma

**DOI:** 10.3390/ijtm5030027

**Published:** 2025-06-29

**Authors:** Trang T. T. Nguyen, Eunhee Yi, Christian E. Badr

**Affiliations:** 1 Ronald O. Perelman Department of Dermatology, New York University Grossman School of Medicine, Laura and Isaac Perlmutter Cancer Center, NYU Langone Health, New York, NY 10016, USA; 2 Department of Physiology, College of Human Medicine, Michigan State University, East Lansing, MI 48824, USA; 3 Department of Neurology, Massachusetts General Hospital, Neuroscience Program, Harvard Medical School, Boston, MA 02129, USA

**Keywords:** glioblastoma, PRMT5 inhibitor, MTAP deletion, MTA, synthetic lethality

## Abstract

Glioblastoma (GBM) is the most aggressive primary brain tumor in adults, characterized by a dismal prognosis and limited therapeutic options. Its highly invasive nature and pronounced intratumoral heterogeneity underscores the urgent need for innovative and targeted therapeutic strategies. One promising approach is synthetic lethality, which exploits cancer-specific genetic vulnerabilities to selectively eliminate tumor cells. A well-characterized example involves the deletion of methylthioadenosine phosphorylase (MTAP), commonly observed in GBM and other malignancies. This review focuses on synthetic lethality targeting protein arginine methyltransferase 5 (PRMT5) in MTAP-deleted GBM. Loss of MTAP leads to the accumulation of methylthioadenosine (MTA), a metabolite that partially inhibits PRMT5, thereby creating a selective vulnerability to PRMT5 inhibition which is used to inhibit the residual function of PRMT5. We critically evaluate preclinical and clinical data on both first- and second-generation PRMT5 inhibitors, with particular emphasis on MTA-cooperative compounds that selectively exploit MTAP deficiency. Despite promising anti-tumor activity in vitro, the clinical efficacy of PRMT5 inhibitors is often limited by the tumor microenvironment, particularly the impact of non-malignant cells that attenuate drug activity. Finally, we explore rational combination strategies that integrate PRMT5 inhibition with existing therapies to enhance clinical outcomes in GBM.

## 1. Introduction

Glioblastoma (GBM) is the most aggressive malignant brain tumor in adults, with a median survival of just 15 months despite the current standard-of-care treatments: surgery, radiation, and chemotherapy [1,2]. The frontline chemotherapy, temozolomide, an oral alkylating agent, provides only limited benefits, improving median survival by approximately three months and showing effectiveness in only a subset of patients [1–4]. The poor prognosis is largely due to the highly invasive and heterogenous nature of GBM and its ability to develop resistance to current therapies over time [4–6]. As a result, there is a pressing need to identify novel therapeutic targets, particularly for patients with specific genetic alterations that are not effectively addressed by existing treatments. The goal is to develop more personalized, effective therapies that selectively eliminate GBM cells while sparing healthy tissue.

One promising therapeutic strategy in cancer treatment is the concept of synthetic lethality, which involves targeting a secondary vulnerability that arises due to an existing genetic defect in cancer cells [7,8]. A well-characterized example involves the co-deletion of cyclin-dependent kinase inhibitor 2A (CDKN2A) (encoding p16INK4a and p14ARF) and methylthioadenosine phosphorylase (MTAP), a genetic alteration observed in approximately 10–15% of all human cancers and at even higher frequencies in specific tumor types, including GBM, mesothelioma, pancreatic adenocarcinoma, non-small cell lung cancer (NSCLC), and cholangiocarcinoma [9–11].

**Figure 1.**
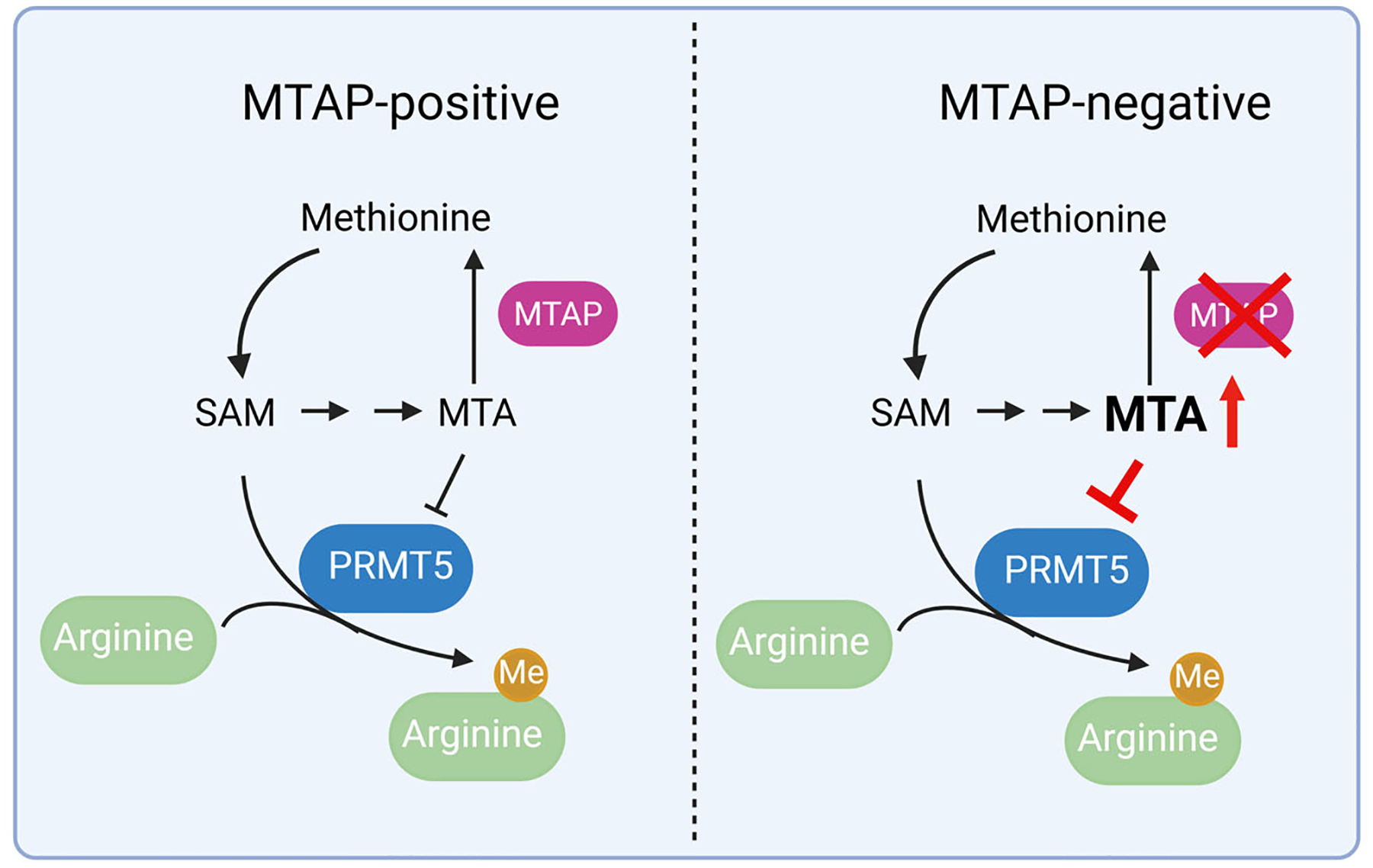
MTAP deletion results in the accumulation of MTA, a metabolite that partially inhibits PRMT5 activity in MTAP-negative cells. SAM: S-adenosyl methionine; MTA: methylthioadenosine; PRMT5: arginine methyltransferase 5; Me: Methylation. Figure created using BioRender.

MTAP is an essential enzyme in the methionine salvage pathway [10,12]. Loss of MTAP leads to the intracellular accumulation of methylthioadenosine (MTA), a metabolite that acts as a natural inhibitor of protein arginine methyltransferase 5 (PRMT5), a key regulator of RNA splicing, transcription, translation, and other vital cellular processes [13,14] (Figure 1). This biochemical consequence has sparked interest in exploiting MTAP loss as a synthetic lethal target. Specifically, MTAP-deleted cancers show reduced basal PRMT5 activity due to MTA accumulation, creating a unique vulnerability that could be therapeutically targeted [9,15].

PRMT5 plays a critical role in supporting tumor cell proliferation and biosynthetic processes [16]. Elevated PRMT5 expression is observed across a wide range of malignancies, including gliomas, melanomas, colorectal carcinomas, leukemias, lymphomas, non-small cell lung cancer (NSCLC), and pancreatic and prostate cancer, and is frequently associated with poor prognosis [8,17]. Functional studies in GBM models have shown that PRMT5 depletion triggers apoptosis or impairs self-renewal, depending on the tumor cells*′* differentiation state [14]. Moreover, *PRMT5* knockout markedly reduced GBM tumor growth in vivo, underscoring a dependency on PRMT5 activity and reinforcing its promise as a druggable target in GBM [17,18]. This review highlights synthetic lethality approaches targeting PRMT5 in MTAP-deleted GBM and explores rational combination strategies that integrate PRMT5 inhibition with existing therapies to improve clinical outcomes in GBM.

## 2. Role of PRMT5 in GBM

Post-translational modifications (PTMs) are essential regulators of protein function, and disruptions in PTM networks are closely associated with cancer development [16,19]. Among these modifications, arginine methylation has emerged as a critical regulatory mechanism in various neoplastic diseases [20]. This PTM is catalyzed by a family of enzymes known as protein arginine methyltransferases (PRMTs), of which nine have been identified in mammals [19,21]. All PRMTs transfer methyl groups from S-adenosylmethionine (SAM) to arginine residues, producing mono- or dimethylated arginine [8,20]. PRMTs are categorized into three types based on the type of methylation they catalyze: Type I enzymes generate asymmetric dimethylarginine (aDMA), Type II enzymes produce symmetric dimethylarginine (sDMA), and Type III enzymes generate only monomethylarginine [14,22].

PRMT5 is the predominant Type II PRMT in mammals, catalyzing the formation of sDMA. It forms a homotetramer and assembles methylosome protein 50 (MEP50/WDR77) to create an active hetero-octameric complex [8,23]. PRMT5 is a pivotal enzyme that orchestrates a wide array of cellular processes, including transcriptional regulation, RNA splicing, signal transduction, and DNA damage repair [24,25]. It carries out these functions, primarily through the symmetric dimethylation of arginine residues on a broad spectrum of substrates, including histones, RNA-binding proteins (RBPs), transcription factors, DNA repair machinery, and various signaling molecules [26,27]. One of the most well-characterized roles of PRMT5 is in epigenetic gene silencing. By symmetrically dimethylating arginine 8 on histone H3 (H3R8me2s) and arginine 3 on histone H4 (H4R3me2s), PRMT5 promotes chromatin condensation and transcriptional repression [23,26,28]. These modifications are crucial for silencing genes involved in cell cycle progression and differentiation, and they play a fundamental role in regulating development, lineage specification, and tumorigenesis [29].

Beyond its chromatin-related functions, PRMT5 is essential for genomic stability [30,31]. It participates in DNA damage response and facilitates homologous recombination repair by methylating core DNA repair proteins [31,32]. Moreover, PRMT5 governs pre-mRNA processing by methylating several components of the spliceosome and RBPs, thereby influencing both constitutive and alternative splicing [24,25,33]. Recent studies have added further complexity to the PRMT5 regulatory network by identifying Chloride Channel Nucleotide-Sensitive 1A (pICln) and Right Open Reading Frame Kinase 1 (RioK1) as mutually exclusive binding partners of the PRMT5/MEP50 complex [31,34–36]. RioK1 and pICln bind to the PRMT5 complex, comprising PRMT5 and WD45/MEP50, in a mutually exclusive manner, forming two distinct assemblies that share a common PRMT5–WD45/MEP50 core. One complex incorporates pICln, while the other includes RioK1. Both pICln and RioK1 act as adaptor proteins, enabling the recruitment of specific substrates, such as the RNA-binding protein nucleolin, to the PRMT5 complex for symmetric arginine methylation [34,36].

PRMT5 is primarily localized in both the nucleus and cytoplasm of GBM cells, with a predominant nuclear presence in many tumor samples [8]. Its nuclear localization is associated with transcriptional repression through the symmetric dimethylation of histone arginine residues (such as H4R3me2s and H3R8me2s), which contributes to the silencing of tumor suppressor genes [8,17,33,37]. Additionally, cytoplasmic PRMT5 has been implicated in the regulation of splicing and translation processes, further supporting its multifaceted role in GBM pathogenesis [24,38]. Splicing is mediated by the spliceosome, a complex of five small nuclear RNPs (snRNPs)—U1, U2, U4, U5, and U6—along with numerous non-snRNP proteins. Each snRNP contains a specific snRNA and a core of seven conserved Sm proteins. In the cytoplasm, Sm proteins are symmetrically dimethylated by the PRMT5-MEP50-pICln methylosome complex, enhancing their binding to the SMN (Survival of Motor Neuron) complex, which removes introns from pre-mRNA during splicing. This modification is essential for proper spliceosome assembly and maintenance of splicing fidelity [39].

## 3. Preclinical and Clinical Studies of PRMT5 Inhibitors in GBM

### 3.1. First-Generation PRMT5-Targeted Therapies

**Figure 2.**
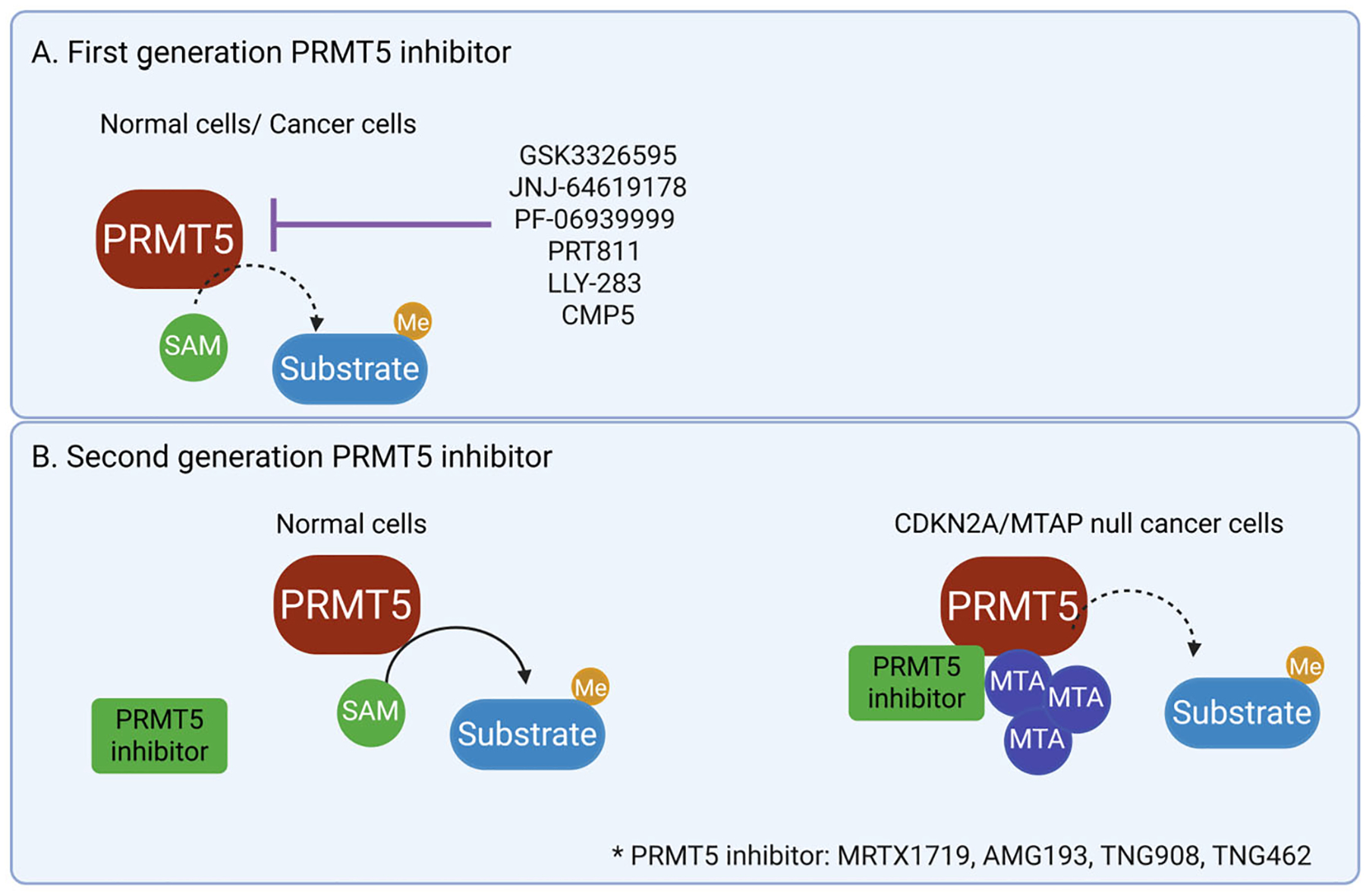
First and second generation of PRMT5 inhibitors. (**A**). First-generation PRMT5 inhibitors directly bind to and inhibit PRMT5 activity in both normal and cancer cells, independent of MTAP status. GSK3326595, JNJ-64619178, PF-06939999, PRT811, LLY-283, CMP5 are PRMT5 inhibitor drugs. (**B**). Second-generation PRMT5 inhibitors exhibit selective efficacy in MTAP-deficient cells, where MTAP loss leads to the accumulation of MTA. The PRMT5 inhibitors only bind to and inhibit PRMT5 in the presence of elevated MTA levels, thereby providing selectivity toward MTAP-null tumors. *PRMT5 inhibitor: MRTX1719, AMG193, TNG908, TNG462. Figure created using BioRender.

Like other methyltransferases, PRMT5 relies on SAM as a methyl group donor [36,40]. Its methyltransferase domain contains two distinct binding pockets: one for SAM and another for the substrate [13]. Early clinical efforts to develop PRMT5 inhibitors focused on broadly suppressing PRMT5 activity, irrespective of the tumor cells*′* MTAP status. These first-generation inhibitors include SAM-competitive PRMT5 inhibitors such as pemrametostat (GSK3326595), as well as dual-site PRMT5 inhibitors that target both the SAM-binding and substrate-binding sites, including onametostat (JNJ-64619178), PRT811, and LLY-283 [30,41–43]. Among them, GSK3326595 and JNJ-64619178 have been studied most extensively. Notably, both compounds demonstrated comparable PRMT5 inhibition in cell lines regardless of MTAP status, showing equal efficacy in MTAP-null and MTAP-wild-type cells [15,22,44]. This finding underscores a significant limitation of these early inhibitors: their inability to selectively target the metabolic vulnerability present in MTAP-deficient tumors (Figure 2).

#### 3.1.1. GSK3326595 (GSK, Philadelphia, PA, USA)

GSK3326595 has shown early promise in preclinical studies and has advanced to clinical evaluation [45]. A Phase I dose-escalation trial (NCT02783300) was initiated to assess the safety, tolerability, pharmacokinetics, and preliminary efficacy of GSK3326595 in patients with advanced solid tumors, including GBM, triple-negative breast cancer (TNBC), metastatic transitional cell carcinoma of the bladder, and non-Hodgkin’s lymphoma [46]. This trial comprises two parts: a dose-escalation phase to establish the recommended Phase 2 dose, followed by a dose-expansion phase to further evaluate safety and efficacy in selected tumor types. Adverse events were common but generally manageable, and clinical responses were observed across multiple malignancies [46].

In Part 1 of the trial, the recommended Phase 2 dose was initially established at 400 mg once daily [9,40,46]. However, following ongoing safety and tolerability assessments, the dose was adjusted to 300 mg once daily. Patients with GBM received treatment for a median duration of 1.4 months (range: 0–7.1 months) [46] (Figure 2 and Table 1).

#### 3.1.2. JNJ-64619178 (Janssen Research & Development, LLC, New Brunswick, NJ, USA)

JNJ-64619178 is currently under development as a therapeutic candidate for advanced malignant solid tumors and lymphomas [15,22]. It functions through simultaneously occupying both the SAM cofactor and protein substrate binding pockets of PRMT5, effectively trapping the PRMT5/MEP50 complex in a catalytically inactive conformation [22]. This dual-site binding results in slow off-rate kinetics and the pseudo-irreversible inhibition of protein arginine dimethylation, enabling sustained and potent target engagement. In preclinical models, JNJ-64619178 induced cancer cell death in a dose-dependent manner, as shown by both in vitro and in vivo drug studies [8,15,44].

A Phase 1, open-label, multicenter study (NCT03573310) was conducted to evaluate the safety, pharmacokinetics, pharmacodynamics, and preliminary efficacy of JNJ-64619178 in patients with advanced solid tumors, non-Hodgkin lymphomas (NHL), and lower-risk myelodysplastic syndromes (MDS) [44]. While preclinical data and early clinical results demonstrated encouraging anti-tumor activity, treatment with JNJ-64619178 has been associated with dose-limiting hematological toxicities, including thrombocytopenia and neutropenia [44]. These adverse effects are likely due to the non-selective inhibition of PRMT5 in normal hematopoietic cells, where PRMT5 plays a critical role in maintaining essential physiological functions (Figure 2 and Table 1).

#### 3.1.3. PF-06939999 (Pfizer, New York, NY, USA)

PF-06939999 was evaluated in a phase I clinical trial (NCT03854227) to assess its safety, tolerability, pharmacokinetics, pharmacodynamics, and anti-tumor activity in patients with advanced solid tumors. In the dose-escalation phase (part 1), 28 patients received oral doses ranging from 0.5 mg once daily to 6 mg twice daily [15,50]. Dose-limiting toxicities were observed in 17% of patients, but no maximum tolerated dose was established. Based on the safety and pharmacodynamic data, the recommended phase 2 dose (RP2D) was determined to be 6 mg once daily [41]. In the dose-expansion phase (part 2), 26 additional patients were treated at RP2D. The most common grade 3 treatment-related adverse events included anemia (28%), thrombocytopenia (22%), fatigue (6%), and neutropenia (4%) [41,50]. Pharmacodynamic assessments showed a dose-dependent reduction in plasma symmetric dimethylarginine (SDMA), supporting target engagement. Clinically, 6.8% of patients achieved a partial response, and 43.2% had stable disease, although no predictive biomarkers for response were identified. Overall, PF-06939999 demonstrated a manageable safety profile and early signs of anti-tumor activity, highlighting PRMT5 as a viable but biologically complex target [41]. However, in April 2022, Pfizer announced the discontinuation of the trial following a strategic portfolio review, citing no safety concerns or regulatory issues in the decision (Figure 2 and Table 1).

#### 3.1.4. PRT811 (Prelude Therapeutics, Wilmington, DE, USA)

PRT811 is a selective PRMT5 inhibitor with the ability to cross the BBB [51]. It is currently being evaluated in a Phase 1, multicenter, open-label, dose-escalation and dose-expansion clinical trial (NCT04089449) in patients with advanced cancers lacking approved or available treatment options [48,50]. Eligible tumor types include solid tumors, central nervous system (CNS) lymphomas, and high-grade gliomas [15].

To date, 45 participants have been enrolled in the ongoing dose-escalation phase, including 18 patients with GBM (n = 17) and anaplastic astrocytoma (n = 1) and 27 patients with advanced solid tumors, such as adenoid cystic carcinoma (n = 4), uveal melanoma (n = 4), acinar cell pancreatic cancer (n = 1), and large-cell neuroendocrine lung cancer (n = 1) [48]. Patients received oral PRT811 at doses ranging from 15 mg to 800 mg in 21-day cycles, administered either continuously or on a 2-week-on/1-week-off schedule [48]. Pharmacokinetic analysis revealed a time to maximum plasma concentration (Tmax) of 1–3 h and a half-life (T1/2) of approximately 5.8 h, with linear pharmacokinetics observed [48]. A durable complete response was achieved in a patient with GBM harboring an isocitrate dehydrogenase 1 (IDH1) mutation (Figure 2 and Table 1).

#### 3.1.5. LLY-283 (Eli Lilly, Indianapolis, IN, USA)

LLY-283 has demonstrated efficient BBB permeability [15,33]. When administered orally, LLY-283 significantly extended survival in mice bearing orthotopic, patient-derived GBM xenografts, a clinically relevant preclinical model, highlighting its therapeutic potential [33]. In vivo pharmacokinetic analyses showed that LLY-283 achieved favorable brain-to-plasma distribution, with brain tissue concentrations nearly twice those in plasma and cerebrospinal fluid.

In vivo studies using orthotopic patient-derived GBM xenograft models demonstrated that oral administration of LLY-283 (50 mg/kg, 3 days on/4 days off) significantly extended median survival to 37 days versus 30 days in vehicle-treated controls [50]. Although LLY-283 is effective, it presents significant dosing challenges. Every-other-day administration caused substantial toxicity, with mice experiencing over 20% body weight loss. In contrast, a schedule of 3 consecutive days followed by 4 days off per week reduced weight loss to below 10%, making it a more tolerable regimen for extended treatment [43,50]. These findings underscore the importance of further preclinical studies to fully assess the therapeutic window and long-term efficacy of PRMT5 inhibition in GBM (Figure 2).

#### 3.1.6. CMP5

CMP5, a compound designed using structure-based drug discovery methods, was developed by leveraging the crystal structure of a highly homologous rat PRMT1 enzyme [18]. This structural framework enabled silico modeling to design small molecules capable of selectively targeting the catalytic site of PRMT5, thereby inhibiting its enzymatic function. Preclinical testing of CMP5 demonstrated encouraging therapeutic potential [18,52,53]. The in vivo administration of CMP5 in a zebrafish model bearing intracranial GBM tumors showed a significant reduction in tumor burden, alongside a notable increase in overall survival [18]. Further mechanistic studies revealed that CMP5 exerts its anti-tumor effects through the induction of apoptosis in differentiated GBM cells [52]. Additionally, in immature primary tumor cells, CMP5 could trigger a senescence-like state, suggesting that the compound not only halts tumor progression but may also reprogram tumor cells into a less proliferative phenotype [18]. Together, these findings support the development of CMP5 as a therapeutic agent that promotes cell death in differentiated cancer cells while inducing senescence in tumor-initiating populations [54] (Figure 2).

### 3.2. Second-Generation PRMT5-Targeted Therapies

Despite the promising biological premise, first-generation PRMT5 inhibitors such as pemrametostat and onametostat have failed to capitalize on this tumor-selective mechanism [42]. These compounds do not exhibit enhanced activity in the presence of MTA and therefore lack the selectivity necessary to spare normal, MTAP-proficient tissues [55,56]. As a result, their clinical application has been limited by toxicity, particularly myelosuppression, which constrains the therapeutic window [22,41,43,46]. This shortcoming has spurred significant interest in developing a new generation of PRMT5 inhibitors that incorporate MTA-cooperative mechanisms of action, compounds specifically designed to exploit the synthetic lethality between MTAP deletion and PRMT5 inhibition [57].

In response to this unmet need, a novel class of MTA-cooperative PRMT5 inhibitors has emerged, demonstrating selective efficacy in the context of MTAP loss [58]. These second-generation inhibitors include MRTX1719, AM-9747 (a lead compound for AMG193), TNG908, TNG462, and AZD3470. Among them, MRTX1719 represents a significant advancement, discovered through structure-based drug design following a fragment-based screening strategy [49,59–62] (Figure 2).

#### 3.2.1. MRTX1719 (BMS-986504-Bristol Myers Squibb, Princeton, NJ, USA)

MRTX1719 demonstrates strong anti-tumor activity in MTAP-null cancer models, both in vitro and in vivo [63]. Preclinical studies show that MRTX1719 significantly reduces levels of sDMA in spliceosomal, transcriptional, and cell cycle-associated proteins specifically in MTAP-deleted cells, while sparing normal tissues [15,60]. In vitro data reveal that MRTX1719 exhibits over 70-fold selectivity in killing MTAP-deficient cells compared to their MTAP-expressing counterparts, highlighting its potential to minimize off-target toxicity and offering a promising therapeutic strategy for genetically defined tumors [64].

Pharmacokinetic studies indicate that MRTX1719 efficiently penetrates the BBB, with peak brain concentrations observed approximately four hours after subcutaneous dosing [65]. The favorable brain-to-plasma concentration ratio underscores its suitability for targeting central nervous system malignancies, including gliomas. A Phase 0/1 clinical trial (NCT06883747) is currently underway to evaluate MRTX1719 in patients with recurrent GBM characterized by confirmed MTAP loss or deletion [60].

**Figure 3.**
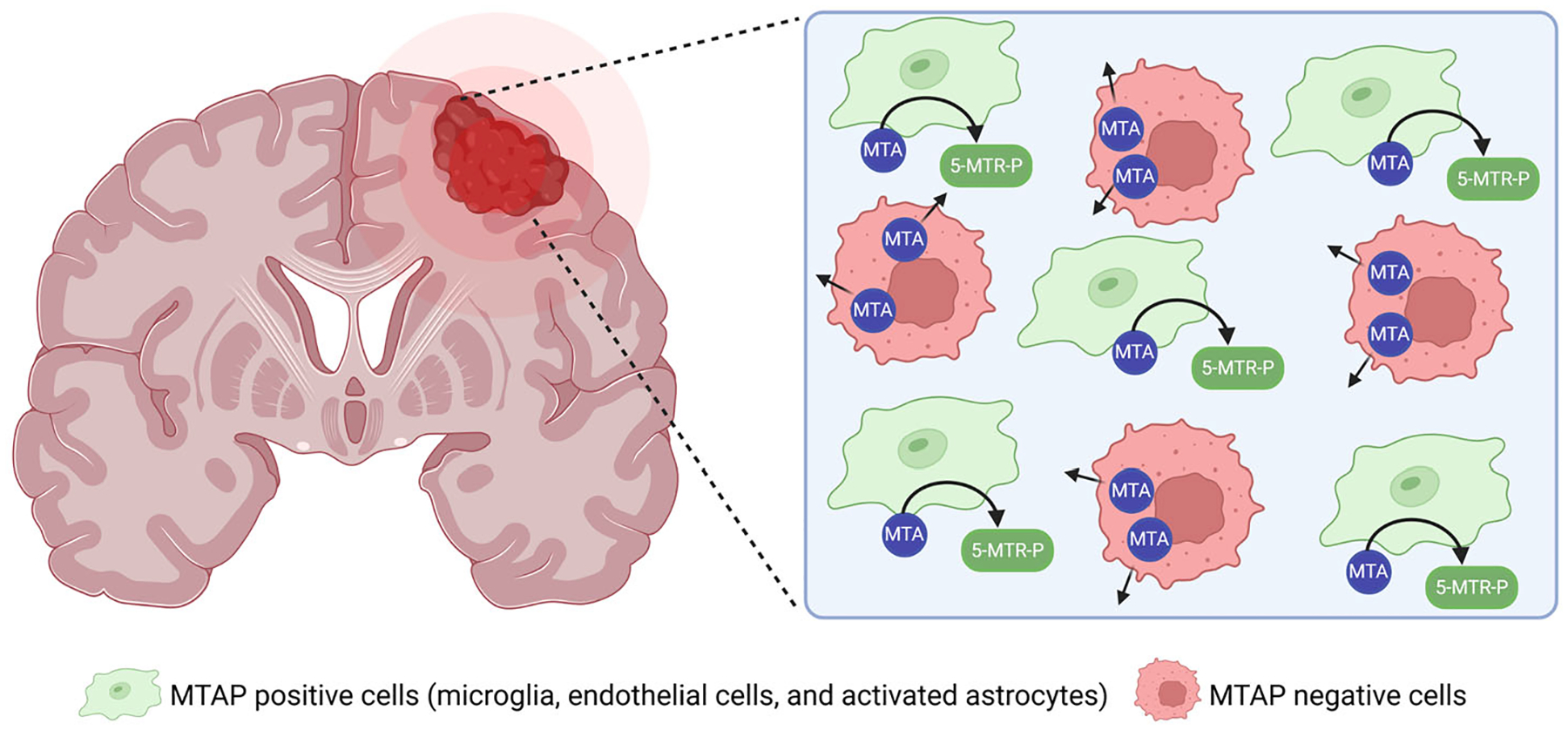
Microenvironment-driven modulation of therapeutic response in MTAP-deficient gliomas. Any MTA secreted by MTAP-deleted glioma cells can be phosphorylated into 5-methylthioadenosine-P (5-MTR-P) and adenine by the MTAP present in surrounding MTAP-expressing cells [58,66]. Figure created using BioRender.

However, while the in vitro efficacy of MRTX1719 against MTAP-deficient glioma cell lines is promising, the in vivo tumor microenvironment presents significant challenges that may hinder therapeutic outcomes. One key factor is the role of surrounding stromal and immune cells that retain normal MTAP expression [64,66]. Although vitro models have shown that MTAP-deficient tumor cells accumulate extracellular MTA due to their inability to metabolize it, in vivo studies have painted a more complex picture. In primary GBM tumors harboring homozygous MTAP deletions, MTA accumulation is not as pronounced [66]. This discrepancy is likely due to the metabolic activity of neighboring non-tumor cells, such as microglia and other stromal components, which are capable of efficiently degrading extracellular MTA [66]. Immunofluorescence staining of glioma tissues supports this hypothesis, revealing that MTAP-deficient tumor regions are often closely surrounded by microglia that express normal levels of MTAP [64]. This spatial proximity suggests a potential metabolic “buffering” effect that may reduce the therapeutic vulnerability of MTAP-deficient glioma cells in vivo (Figures 2 and 3, and Table 1).

#### 3.2.2. AMG193 (Amgen, Thousand Oaks, CA, USA)

AMG193 displays a remarkable approximately 60-fold increase in binding affinity for PRMT5 in the presence of MTA [67]. AMG193 exhibits strong selectivity for MTAP-deleted cancer cell lines, with sensitivity tightly linked to *PRMT5* genetic dependency [15]. Mechanistic studies reveal that AMG193 disrupts RNA splicing machinery, induces cell cycle arrest, and triggers DNA damage in sensitive tumor models [62].

Phase I clinical data for AMG193 demonstrated a favorable safety profile, with no dose-limiting myelosuppression, unlike earlier non-selective PRMT5 inhibitors [61]. A total of 80 patients received AMG193 at doses ranging from 40 to 1600 mg (once daily) or 600 mg (twice daily) in a treatment cycle of 28 days. The most common treatment-related side effects were nausea (48.8%), fatigue (31.3%), and vomiting (30.0%). The maximum tolerated dose was 1200 mg once daily, with AMG 193 exposure increasing proportionally from 40 mg to 1200 mg. Among 42 efficacy-assessable patients treated at active doses (800 mg once daily, 1200 mg once daily, or 600 mg twice daily), the objective response rate was 21.4%, with responses in multiple tumor types, including lung cancer, pancreatic adenocarcinoma, and biliary tract cancer [61].

In a cohort of patients with pancreatic ductal adenocarcinoma (PDAC) treated at biologically active dose levels, a partial response was achieved, indicating initial clinical efficacy [63,68]. Building on these results, AMG193 is now being evaluated in combination with standard chemotherapy regimens in PDAC as part of an ongoing clinical trial (NCT06360354) (Figure 2).

#### 3.2.3 TNG908 and TNG462 (Tango Therapeutics, Boston, MA, USA)

TNG908 is a BBB-penetrant compound with a GI_50_ of less than 10 nM in MTAP-null cells, over 30-fold selectivity versus MTAP wild-type cells, and an extended predicted human half-life (T_1/2_) to support once-daily dosing [69,70]. Preclinical studies have shown that TNG908 induces potent tumor growth inhibition, including sustained regressions and complete responses across a variety of MTAP-deleted xenograft models representing multiple tumor types [15]. TNG908 is currently being evaluated in a Phase I/II clinical trial (NCT05275478) to assess its safety, tolerability, pharmacokinetics, and preliminary ant-tumor activity in patients with advanced or metastatic MTAP-deleted solid tumors, including GBM [49]. This clinical program highlights the therapeutic promise of targeting PRMT5 in genetically defined patient populations.

Following the selection of TNG908 as a development candidate, Tango Therapeutics advanced their efforts to develop TNG462, a second-generation compound with significantly improved potency, selectivity, and pharmacokinetic properties compared to TNG908 [69,71]. TNG462 demonstrates a biochemical potency below the limit of assay detection, with an estimated GI_50_ of 4 nM in MTAP-null cells and 45-fold selectivity over MTAP wild-type cells. It also displays consistent pharmacokinetic properties across preclinical species, with a predicted human half-life exceeding 24 h, enabling once-daily dosing. TNG462 is currently in Phase I/II clinical trials for MTAP-deleted cancers (NCT05732831) [69] (Figure 2 and Table 1).

Collectively, these advances highlight a shift toward precision-targeted PRMT5 inhibition strategies that exploit MTAP loss, a prevalent deletion in many human cancers, as a tumor-selective vulnerability. The development of MTA-cooperative PRMT5 inhibitors marks a promising step forward in achieving potent anti-tumor activity with reduced systemic toxicity.

## 4. Combination Strategies with PRMT5 Inhibitors in GBM Therapy

Although targeting PRMT5 is a validated synthetic lethal approach in MTAP-deficient cancers, combination strategies are crucial to enhance therapeutic efficacy [72,73]. Singleagent PRMT5 inhibitors often lead to incomplete responses and eventual resistance due to tumor heterogeneity and the activation of compensatory pathways [72]. Combining PRMT5 inhibition with agents targeting parallel or downstream vulnerabilities can improve response durability, suppress resistance mechanisms, and potentially reduce toxicity by allowing lower dosing [59,72–74]. Such strategies aim to maximize synthetic lethality and broaden therapeutic impact across diverse MTAP-deficient tumor contexts.

### 4.1. Combining PRMT5 Inhibition with Irradiation

JNJ-64619178 has been shown to inhibit key genes involved in the repair of doublestrand breaks (DSBs) through both homologous recombination (HR) and non-homologous end joining (NHEJ) pathways [32]. These repair mechanisms are essential for maintaining genomic integrity, particularly after DNA damage induced by ionizing radiation (IR) [24]. Cancer cells, especially in malignancies such as GBM, prostate cancer, and lung cancer, often exhibit increased radioresistance. This resistance is typically attributed to the cancer cell′ ability to efficiently repair IR-induced DNA damage, allowing tumors to evade the lethal effects of radiation therapy [75].

Recent research demonstrated that treatment with JNJ-64619178 significantly reduced colony formation in both A549 (lung cancer) and U87-MG (GBM) cell lines exposed to IR, compared to vehicle (DMSO)-treated controls [30]. The reduction in colony number in the JNJ-64619178-treated groups indicates that the inhibition of PRMT5 sensitized these cancer cells to the cytotoxic effects of radiation. This suggests that JNJ-64619178 can enhance the therapeutic efficacy of IR by disrupting the DNA repair mechanisms, specifically targeting PRMT5 [30]. These findings provide strong evidence that PRMT5 inhibition holds promise as a strategy to overcome radioresistance and improve the effectiveness of radiation therapy in cancer treatment.

### 4.2. Combining PRMT5 Inhibition with CDK4/6 Inhibitor

CDK4/6 activity is often elevated in CDKN2A-deleted cells because CDKN2A encodes the tumor suppressor p16INK4a, which is a negative regulator of CDK4 and CDK6 [76]. When CDKN2A is deleted or inactivated, p16INK4a is lost, resulting in unchecked activation of CDK4/6, which promotes cell cycle progression from G1 to S phase via phosphorylation of the retinoblastoma protein (pRB) [77]. Although CDK4/6 inhibitors have been tested in CDKN2A-deleted cells in vitro, they have yet to demonstrate curative efficacy in clinical settings [76,78]. This limited success may be attributed to the elevated CDK4/6 activity commonly observed in CDKN2A-deleted cells.

Nearly all MTAP-deleted cancers also harbor the co-deletion of CDKN2A, as MTAP loss typically occurs as a passenger event alongside CDKN2A deletion [9,10,76]. The loss of CDKN2A has been shown to enhance sensitivity to CDK4/6 inhibition across various cancer cell lines. The combination of TNG908 (a PRMT5 inhibitor) with CDK4/6 inhibitors (like abemaciclib or palbociclib) shows clear efficacy in xenograft models, regardless of individual drug sensitivity [49,78]. This synergistic effect was further validated in vivo using MTAP-null xenograft models of NSCLC and GBM.

### 4.3. Combining PRMT5 Inhibition with MEK Inhibitor

The RAS-RAF-MEK-ERK (mitogen-activated protein kinase/ERK kinase)/ERK (extracellular-signal-regulated kinase) signaling pathway is one of the most commonly dysregulated cascades in cancer and plays a critical role in tumorigenesis [79,80]. In GBM, although RAF mutations are rare, tumor cell proliferation remains highly dependent on elevated MEK-ERK activity. Trametinib, an FDA-approved MEK inhibitor, is currently under clinical investigation for various cancers, including high-grade gliomas and pediatric treatment-refractory central nervous system tumors [81–83]. Despite initial effectiveness, GBM tumor cells eventually develop resistance to trametinib, primarily due to the activation of compensatory survival pathways [84–87]. Notably, the inhibition of *PRMT5* has been shown to sensitize GSC to trametinib by enhancing drug-induced apoptosis [88]. Mechanistically, trametinib-induced AKT activation is suppressed by *PRMT5* knockdown, suggesting that PRMT5 regulates ERK signaling via the PI3K-AKT axis [88]. In vivo, the combination of *PRMT5* inhibition with trametinib significantly reduces tumor growth and prolongs survival [88]. These findings highlight *PRMT5* inhibition as a promising strategy to overcome trametinib resistance in GBM.

### 4.4. Combining PRMT5 Inhibition with PTEN Deficiency in Cancer Therapy

Phosphatase and tensin homolog (PTEN) plays a critical tumor-suppressive role in GBM by negatively regulating the phosphatidylinositol 3-kinase (PI3K)/AKT signaling pathway, a central driver of tumor growth, survival, and resistance to therapy [89,90]. In GBM, AKT is frequently hyperactivated, making it a key target for drug development. PTEN loss leads to sustained AKT activation, which promotes oncogenic signaling and contributes to GBM progression [90–92]. Studies have shown that *PRMT5* inhibition induces synthetic lethality in PTEN-null cancers. In other words, while loss of PTEN or inhibition of *PRMT5* alone might not be fatal, their combination (loss of PTEN and inhibition of *PRMT5*) is lethal to cancer cells. This suggests *PRMT5* acts as a survival buffer in PTEN-deficient settings [91].

### 4.5. Combining PRMT5 Inhibition with mTOR Inhibitor

The mammalian target of rapamycin (mTOR) is a central protein kinase that governs critical cellular processes, including growth, proliferation, metabolism, protein synthesis, autophagy, and energy homeostasis [93–95]. mTOR primarily exerts its function through the activation of mTOR complex 1 (mTORC1) via the PI3K/Akt signaling pathway [91,96,97]. In GBM, mTOR inhibition has been found to modulate PRMT5 activity in a complex, nontranscriptional manner [17,91]. Specifically, pharmacologic inhibition of mTOR results in a linear increase in PRMT5 enzymatic activity without changes in its mRNA or protein expression, suggesting regulation at the post-translational or indirect level [98].

The combination of mTORC1/2 inhibitors with EPZ015666, a selective PRMT5 inhibitor, demonstrated a synergistic effect in GBM cells, enhancing apoptotic cell death [98]. This dual treatment also led to the upregulation of oncogenes such as cyclin D1 and c-MYC, key drivers of cell cycle progression and transformation, indicating a potential disruption of cell cycle checkpoints that sensitize cells to apoptosis [98].

These synergistic effects were corroborated in vivo using the LN229 GBM xenograft model. Mice receiving the combination therapy exhibited a marked 75% reduction in tumor growth, significantly surpassing the effects of single-agent treatments, which achieved only 30% (EPZ015666) and 29% (mTORC1/2 inhibitors) tumor inhibition, respectively [98]. Collectively, these findings highlight the promise of co-targeting mTOR and PRMT5 as a strategy to enhance anti-tumor efficacy and overcome resistance mechanisms that limit the success of monotherapies in GBM.

### 4.6. Combining PRMT5 Inhibition with PP2A Inhibitor

Protein Phosphatase 2A (PP2A) is a tumor suppressor that plays a critical role in regulating cellular senescence, a process linked to treatment resistance in cancer [99]. LB100, a first-in-class small-molecule inhibitor of PP2A, has demonstrated the ability to sensitize tumors to chemotherapy and radiation [100]. In a completed phase 1 trial (NCT01837667), LB100 was well-tolerated in adults with progressive solid tumors and is now being evaluated in phase 2 trials for GBM (NCT03027388) and myelodysplastic syndromes (NCT03886662). Notably, combining sublethal doses of LB100 with PRMT5 inhibition (via genetic deletion or JNJ-64619178) enhances necroptosis, leading to improved anti-tumor efficacy [99]. This combinatorial strategy, supported by the favorable safety profile of LB100, represents a promising therapeutic approach to augment the effects of PRMT5 inhibition in GBM.

### 4.7. Combining PRMT5 Inhibition with PD-1 Inhibitor

In a lung cancer study, PRMT5 inhibitors like GSK3326595 demonstrated a selectively targeting and killing cancer cells, but also potentially weakening the anti-tumor immune response by increasing PD-L1 (CD274) expression, which suppresses CD8+ T cell function [42]. Mechanistically, PRMT5 regulates CD274 gene expression by catalyzing the symmetric dimethylation of histone H4R3, leading to increased H3R4me2s deposition at the CD274 promoter and suppression of its transcription. The inhibition of PRMT5 disrupts this repressive chromatin state, resulting in the upregulation of CD274 expression in lung cancer cells. A study showed that combination PRMT5/PD-L1 inhibition may achieve a better anti-tumor effect: targeting PRMT5 to directly inhibit tumor cell survival and targeting PD-L1 to overcome the side effects of targeting PRMT5 and enhance immune function [42,50].

NCT02783300 is a Phase I open-label, dose-escalation study designed to evaluate the safety, pharmacokinetics, pharmacodynamics, and clinical activity of GSK3326595 in patients with advanced or recurrent solid tumors and non-Hodgkin′s lymphoma [46]. Part 3 of the study specifically investigated the combination of GSK3326595 with pembrolizumab in selected solid tumors, including triple-negative breast cancer, GBM, and NSCLC [46].

## 5. Conclusions and Future Perspectives

Although PRMT5 inhibitors remain a promising therapeutic strategy, a significant unmet need lies in identifying the tumor types and patient subpopulations that are most likely to benefit from this approach. Achieving this goal requires a thorough understanding of both the clinical prevalence and molecular landscape of MTAP deletions across diverse cancer types. While MTAP loss is frequent in several aggressive malignancies including gliomas, the therapeutic translation of these findings into effective in vivo treatments has proven challenging. For example, while PRMT5 inhibitors such as MRTX1719 exhibit strong anti-tumor activity in MTAP-deficient glioma cells in vitro, their efficacy in vivo is attenuated by the influence of the TME [64,66]. One key factor contributing to this resistance is the presence of non-malignant, MTAP-expressing cells within the TME, such as microglia. These cells are capable of metabolizing extracellular MTA, thereby reducing its local concentration and attenuating the inhibitory effects on PRMT5 (Figure 3). Moreover, the extent to which different cell types within the TME can modulate MTA levels through active efflux or enzymatic degradation remains poorly understood. Elucidating these mechanisms, particularly the regulation of MTA transport and metabolism, could provide critical insights into overcoming microenvironment-driven resistance to PRMT5-targeted therapies. Inhibiting MTA transport may enhance the efficacy of PRMT5 inhibitors by preventing the MTA-mediated suppression of drug activity.

To improve therapeutic outcomes, rational combination strategies should be pursued. These may include approaches that reprogram the TME to reduce MTA clearance, inhibit compensatory metabolic pathways, or selectively enhance the accumulation of MTA within the tumor. Additionally, combining PRMT5 inhibitors with agents that act synergistically, such as CDK inhibitors, immune checkpoint blockers, or modulators of methylation-dependent gene expression, may further boost anti-tumor efficacy while minimizing off-target effects. Ultimately, integrating molecular diagnostics with targeted therapies offers great promise for realizing the full potential of PRMT5 inhibition in MTAP-deficient cancers, including GBM, as well as other tumors with high MTAP deletion frequencies, such as mesothelioma, pancreatic adenocarcinoma, NSCLC, and cholangiocarcinoma.

## Figures and Tables

**Table 1.** Current and completed clinical trials of PRMT5 inhibitors in GBM from ClinicalTrials.gov.

Clinical Trial Identifier/Therapy	Study Name	Phase/Recruitment Status	Key Findings/Conclusions
NCT02783300/GSK3326595	An Open-label, Dose Escalation Study to Investigate the Safety, Pharmacokinetics, Pharmacodynamics and Clinical Activity of GSK3326595 in Participants with Solid Tumors and Non-Hodgkin′s Lymphoma (Meteor 1)	Phase 1/completed	GSK3326595 was generally well-tolerated, with about 95% of patients experiencing treatment-related adverse events, most commonly fatigue, anemia, nausea, and alopecia [46].
NCT03573310/JNJ-64619178	A Study of JNJ-64619178, an Inhibitor of PRMT5 in Participants with Advanced Solid Tumors, NHL, and Lower Risk MDS	Phase 1/active, not recruiting	JNJ-64619178 showed manageable safety, early anti-tumor activity, and suitable dosing, supporting further evaluation in Phase 2 trials [22,47].
NCT03854227/PF-06939999	A Dose Escalation Study Of PF-06939999 In Participants with Advanced or Metastatic Solid Tumors	Phase 1/terminated	PF-06939999 was generally well-tolerated. Dose-limiting toxicities were observed in 17% of patients during the dose-escalation phase, including thrombocytopenia, anemia, and neutropenia [41].
NCT04089449/PRT811	A Study of PRT811 in Participants with Advanced Solid Tumors, CNS Lymphoma and Gliomas	Phase 1/completed	PRT811 demonstrated an acceptable safety profile [48].
NCT06883747/BMS-986504 (MRTX1719)	Clinical Trial of BMS-986504 in Recurrent GBM Patients	Early Phase 1/recruiting	MRTX1719 is being evaluated for its pharmacokinetics (PK), safety, and tolerability in patients with recurrent glioblastoma who harbor MTAP gene deletions.
NCT05275478/TNG908	Safety and Tolerability of TNG908 in Patients With MTAP-deleted Solid Tumors	Phase 1, 2/active, not recruiting	TNG908 is being evaluated in a phase 1/2 clinical trial to assess its safety, tolerability, and early signs of ant-tumor activity in patients with MTAP-deleted advanced or metastatic solid tumors [49].
NCT05732831/TNG462	Safety and Tolerability of TNG462 in Patients With MTAP-deleted Solid Tumors	Phase 1, 2/recruiting	TNG462 is being evaluated in a phase 1/2 clinical trial to assess its safety and tolerability as a single agent and in combination in patients with advanced or metastatic solid tumors harboring MTAP deletions.
